# Quantifying the test–retest reliability of cerebral blood flow measurements in a clinical model of on-going post-surgical pain: A study using pseudo-continuous arterial spin labelling^☆^

**DOI:** 10.1016/j.nicl.2013.09.004

**Published:** 2013-09-16

**Authors:** Duncan J. Hodkinson, Kristina Krause, Nadine Khawaja, Tara F. Renton, John P. Huggins, William Vennart, Michael A. Thacker, Mitul A. Mehta, Fernando O. Zelaya, Steven C.R. Williams, Matthew A. Howard

**Affiliations:** a Centre for Neuroimaging Sciences, Institute of Psychiatry, Kings College London, London, UK; b MRC Social, Genetic and Developmental Psychiatry Centre, Institute of Psychiatry, Kings College London, London, UK; c Kings College London Dental Institute, London, UK; d Global Research and Development, Pfizer Limited, Sandwich, Kent, UK

**Keywords:** ASL, CBF, ICC, Reliability, Test–retest, Pain, VAS

## Abstract

Arterial spin labelling (ASL) is increasingly being applied to study the cerebral response to pain in both experimental human models and patients with persistent pain. Despite its advantages, scanning time and reliability remain important issues in the clinical applicability of ASL. Here we present the test–retest analysis of concurrent pseudo-continuous ASL (pCASL) and visual analogue scale (VAS), in a clinical model of on-going pain following third molar extraction (TME). Using ICC performance measures, we were able to quantify the reliability of the post-surgical pain state and ΔCBF (change in CBF), both at the group and individual case level. Within-subject, the inter- and intra-session reliability of the post-surgical pain state was ranked good-to-excellent (ICC > 0.6) across both pCASL and VAS modalities. The parameter ΔCBF (change in CBF between pre- and post-surgical states) performed reliably (ICC > 0.4), provided that a single baseline condition (or the mean of more than one baseline) was used for subtraction. Between-subjects, the pCASL measurements in the post-surgical pain state and ΔCBF were both characterised as reliable (ICC > 0.4). However, the subjective VAS pain ratings demonstrated a significant contribution of pain state variability, which suggests diminished utility for interindividual comparisons. These analyses indicate that the pCASL imaging technique has considerable potential for the comparison of within- and between-subjects differences associated with pain-induced state changes and baseline differences in regional CBF. They also suggest that differences in baseline perfusion and functional lateralisation characteristics may play an important role in the overall reliability of the estimated changes in CBF. Repeated measures designs have the important advantage that they provide good reliability for comparing condition effects because all sources of variability between subjects are excluded from the experimental error. The ability to elicit reliable neural correlates of on-going pain using quantitative perfusion imaging may help support the conclusions derived from subjective self-report.

## Highlights

• Test-retest reliability of pCASL is considered in a post-surgical pain model.
• Pain-state and ∆CBF measurements were reliable at the group and individual level.
• Single or average baseline measurements improve reliability of ∆CBF.
• pCASL is a reliable technique for detecting cerebral responses to on-going pain.

## 1 Introduction

Pain is a complex, multidimensional experience that includes sensory and affective components. Within this context, pain is subjective and is not readily quantifiable. For humans, pain assessment strategies may include self-rating scales, observational scales, and other behavioural tools (Katz and Melzack, 1999). One of the most commonly used methods for assessing pain in the clinic is the visual analogue scale (VAS). While this assessment is by definition, highly subjective, these scales are of most value when looking at changes within individuals, and are of less value for comparing across a group of individuals at one particular time (Steingrimsdottir et al., 2004; Victor et al., 2008). Critically, there is an acknowledged, unmet need for more reliable endpoints of the pain experience (Kupers and Kehlet, 2006). The identification of robust and quantifiable measurement tools is likely to improve the diagnosis and management of chronic pain conditions, and help provide a better evaluation of the mechanisms of analgesic drugs.

Neuroimaging techniques have demonstrated that a large, distributed brain network underpins nociceptive processing. In the past, authors have referred to this network as the “pain matrix” (Brooks and Tracey, 2005); however this concept has been challenged, as relevant salient or behavioural stimuli have been shown to engage a similar network (Downar et al., 2003; Iannetti and Mouraux, 2010). For acute pain experiences, commonly activated areas include the primary and secondary somatosensory cortices, insular, anterior cingulate, prefrontal cortex, and the thalamus (Apkarian et al., 2005; Tracey and Bushnell, 2009). Depending on the nociceptive stimulus and experimental paradigm, other brain regions including the basal ganglia, cerebellum, amygdalae, hippocampus, and areas within the parietal and temporal cortices may also be recruited. By contrast, the mechanisms that contribute to the generation and maintenance of chronic clinical pain states are more complex. Several groups have reported consistent activation in the prefrontal, frontal, and anterior insular cortices that may be important in the maintenance of chronic pain conditions (Apkarian et al., 2009; Howard et al., 2012; Schweinhardt and Bushnell, 2010; Wasan et al., 2011). However, it is still unclear if these markers of activity directly predict the underlying clinical pathology, or represent other contextual aspects of the patients' experiences.

Owing to the advent of arterial spin labelling (ASL) MRI techniques, the representation of on-going or spontaneous pain states has rightly received attention in neuroimaging (Howard et al., 2011; Maleki et al., 2013; Owen et al., 2008, 2010; Tracey and Johns, 2010). Our group recently reported a study using pseudo-continuous ASL (pCASL) (Dai et al., 2008), in conjunction with a commonly used post-surgical model, to demonstrate changes in regional cerebral blood flow (CBF) associated with the experience of being in on-going pain after third molar extraction (TME) (Howard et al., 2011). This study identified a number of the anatomical regions consistent with pain response patterns detected using ASL in other experiments (reviewed in Maleki et al., 2013). Pain following TME has become the most frequently used model in acute pain trials, particularly for regulatory purposes (Barden et al., 2004). However, in the present literature, there is limited information available on the reliability of quantitative perfusion measures for the study of on-going pain in experimental volunteers and patients using ASL methodologies.

A well-established measure of reliability is the intra-class correlation coefficient (ICC) (Shrout and Fleiss, 1979). ICC has classically been described in the context of consistency or agreement between ratings given by different judges; however, it can also be used to assess the reliability of ratings across different testing sessions and to assess the reliability of imaging methods over time (Bennett and Miller, 2010; Caceres et al., 2009). Several groups have conducted reliability studies of resting CBF measurements employing different ASL labelling schemes (Cavusoglu et al., 2009; Chen et al., 2011; Floyd et al., 2003; Gevers et al., 2009, 2011; Hermes et al., 2007; Jahng et al., 2005; Jain et al., 2012; Jiang et al., 2010; Parkes et al., 2004; Petersen et al., 2010; Tjandra et al., 2005; Xu et al., 2010; Yen et al., 2002). These studies converge on the conclusion that ASL reliability is comparable to other perfusion imaging techniques such as PET or SPECT; however, the extracted CBF values are often constrained to the cortical grey matter (GM), flow territories, brain lobes, or targeted regions-of-interest (ROIs). Two recent studies assessed the feasibility of ASL for pharmacological research, conducting test–retest evaluations of citalopram and fentanyl drug challenges (Klomp et al., 2012; Zelaya et al., 2012). To our knowledge, there have been no reports confirming the reliability of ASL-based perfusion measurements for the study of on-going pain states in experimental volunteers or chronic pain patients. Similarly, there have been no ‘head-to-head’ comparisons of the ASL technique with traditional behavioural assessments of pain.

To confidently compare CBF values across different cohorts of a population (i.e. pain patients vs. healthy controls) and across repeated measurements on the same individual (such as in longitudinal cross-over studies and drug trials), it is important to consider the between- and within-subject variability. In this study, we sought to quantify the test–retest reliability of concurrent pCASL and VAS in a clinical model of on-going pain following TME. Reliability was examined at three levels; (1) inter-subject, (2) inter-session, and (3) intra-session. Within each of these categories, we calculated the ICCs for the pre- and post-surgical states, together with the change in CBF (∆CBF) between conditions. The principal aim of this work was to inform on the reliability of the pCASL technique versus VAS subjective pain ratings, and help provide a framework to support future use of ASL methodologies for the study of chronic pain conditions and experimental ongoing pain states.

## 2 Methods

### 2.1 Ethical approval and consent

All procedures were approved by the Kings College Hospital Research Ethics Committee (REC Reference 07/H0808/115). Informed, written consent was provided by all participants.

### 2.2 Inclusion criteria

Sixteen right-handed, healthy male volunteers (age range: 18–50 years) were selected for the study. Participants presented with bilateral recurrent pericoronitis and fulfilled NICE guidelines for extraction of lower-jaw left and right third molars (NICE/NHS, 2000). Females were not included in the study due to potential variability in the phase of the menstrual cycle affecting reproducibility of the post-surgical pain (Teepker et al., 2010).

### 2.3 Study design

**Fig. 1:**
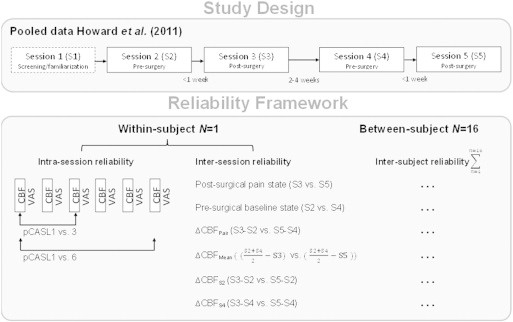
Study design for the assessment of reliability of the pCASL and VAS modalities in the clinical model of on-going post-surgical pain. The data was pooled from two pre- and post-surgical visits to assess group-level inter-subject consistency, and the within-subject inter- and intra-session reliability.

Data were pooled from the previously published work of Howard et al. (2011). Briefly, sixteen subjects were assessed on five separate occasions, screening/familiarisation (S1), pre-surgical scan (S2), post-surgical scan following the first tooth extraction (S3), pre-surgical scan (S4), and postsurgical scan following the second tooth extraction (S5) (Fig. 1). Scanning commenced at S3 and S5 when three consecutive VAS scores greater than 30/100 mm were provided within a 30-minute period. Order of left and right tooth extraction was balanced and pseudo-randomised across the group. A minimum of two week interval separated S3/S4, and participants were assessed based on individual report of pain cessation to ensure complete recovery from the surgery. The rescue medication of 1000 mg paracetamol/400 mg ibuprofen was provided to participants immediately following scanning during S3 & S5. Full alcohol and drug-screens were performed at every visit, including psychometric assessment.

### 2.4 Perfusion MRI

Participants were scanned on a 3 T whole-body MRI scanner (GE Signa HDX) fitted with a receive-only 8-channel, phased-array head coil. For image registration purposes, a high resolution T2-weighted Fast Spin Echo (FSE) image was acquired. Perfusion measurements were made using a pseudo-continuous arterial spin labelling (pCASL) sequence (Dai et al., 2008). Labelling was performed using a train of Hanning RF pulses; 500 μs duration, peak-to-peak gap 1500 μs, and a total labelling duration of 1.5 s. After a post-labelling delay of 1.5 s, the image was acquired with a 3D FSE inter-leaved spiral readout (8 shots, TE/TR = 32/5500 ms, ETL = 64, 3 tag–control pairs). Pre-saturation of the image volume, followed by selective inversion pulses for background suppression, was also acquired in order to minimise the static signal. Two reference images (fluid suppressed and both fluid and white matter suppressed); as well as a coil sensitivity map, were used for the computation of the CBF maps in physiological units (ml blood per 100 g of tissue per min). The ASL time series comprised 6 pCASL scans, lasting 6 min each. Participants were instructed to lie still with their eyes open. Full details of the pCASL sequence and absolute quantification of CBF are available in Supplementary information.

### 2.5 Visual analogue scales

Concurrent with the MRI examination, subjects were asked to rate their perceived levels of pain and alertness using a visual analogue scale (VAS). The VAS measurements were performed according to an established protocol (Howard et al., 2011) which consisted of a computerised line anchored with “no pain”/“worst imaginable pain” and “very sleepy”/“wide awake”. Participants subjectively rated their experience following each of the six pCASL scans using a computerised VAS and button-box.

### 2.6 Image pre-processing

The quantitative CBF data were pre-processed using FSL (http://www.fmrib.ox.ac.uk/fsl) (Smith et al., 2004). The pipeline consisted of skull stripping [BET], affine registration of each subject's T2 to the Montreal Neurological Institute (MNI) ICBM152 non-linear asymmetric T2-weighted template with resampling to 2 × 2 × 2 mm^3^ [FLIRT], and non-linear noise reduction [SUSAN: λ = 5 mm full-width half maximum]. Statistical analysis was performed under the framework of the general linear model (GLM) [FLAMEO]. First-level analyses were computed for each subject to create grey-matter (GM) only mean images of the six individual pCASL scans acquired at each of the sessions S2–S5. For the second-level analysis, changes in the CBF relating to post-surgical pain were obtained using a mixed-effects two-way ANOVA of the combined session-pairs (i.e. Pair 1[S2,S3]/Pair 2[S4,S5]) and a *t*-threshold equivalent to *p* < 0.01 (*z* = 2.3, *t* = 2.41, *dof* = 45). Factorial designs are powerful because the interaction between various cognitive components (factors) is explicitly modelled in the analyses (Friston et al., 1996). However, an anticipated problem with calculating the change in CBF between pre- and post-surgical states (∆CBF) is that arithmetic subtraction between these two conditions will not take account of the error variance. To examine these effects, images of ∆CBF (change in CBF) were calculated in four separate ways: (1) arithmetic subtraction of the pre- and post-surgical session-pairs (∆CBF_Pairs_), (2) subtraction of the post-surgical sessions from the combined mean of the pre-surgery sessions (∆CBF_Mean_), (3) subtraction of the post-surgical sessions from the first pre-surgery session only (∆CBF_S2_), and (4) subtraction of the post-surgical sessions from the second pre-surgery session only (∆CBF_S4_). The same contrast images, for the pre- and post-surgical sessions only, were used to extract the reliability of the independent states (see Fig. 1).

### 2.7 Regions of interest

To assess CBF reliability between subjects and sessions, regions of interest (ROIs) were defined a priori based upon previously implicated areas in pain processing measured with arterial spin labelling (reviewed in Maleki et al. (2013)). ROIs were anatomically defined in standard MNI space from the Harvard–Oxford cortical and subcortical structural atlases, with probabilistic images thresholded at 20% and binarized to create exclusive ROI masks. These were anterior cingulate cortex (ACC), posterior cingulate cortex (PCC), anterior insula (aINS), posterior insula (pINS), somatosensory cortex (primary, S1 and secondary, S2), thalamus (THAL), hippocampus (HIP), amygdala (AMY), and brainstem (BS).

### 2.8 Statistical methods

To systematically evaluate the test–retest performance of the TME post-surgical pain model, we examined the inter-subject, inter-session, and intra-session variability of CBF and VAS measurements (Fig. 1). These reliability estimates were calculated using the third ICC defined by (Shrout and Fleiss, 1979)

(1) $$\mathrm{ICC}\left(3,1\right)=\frac{\mathrm{BMS}-\mathrm{EMS}}{\mathrm{BMS}+\left(k-1\right)\mathrm{EMS}}$$

where BMS is the between-targets mean square, EMS is the error mean square, and k is the number of repeated sessions (here two). All ICC values were calculated in MATLAB 7.1 (The Mathworks Inc.) and the statistical toolbox produced by Caceres et al. (2009) (ICC Toolbox is available for download at: http://www.kcl.ac.uk/iop/depts/neuroimaging/research/imaginganalysis/Software/ICC-Toolbox.aspx). We denote ICC values < 0.4 as poor, 0.4–0.59 as fair, 0.60–0.74 as good, and > 0.75 as excellent (Fleiss et al., 2003). However, these ranges should be interpreted with caution as they do not take into account the confidence intervals of the ICC.

Coefficient of variation (CV) is defined as the ratio of the standard deviation σ to the mean $\sigma :=\frac{\sigma }{\mu }$.

### 2.9 Reliability of the behavioural measures

We examined behavioural changes using the VAS self-report of subjective alertness and pain. Inter-subject consistency was compared using all ratings from the post-surgical pain sessions. Within-subjects the VAS measurements from left and right-side post-surgical pain sessions were used to assess inter-session reliability. Intra-session stability was evaluated using the six VAS measures from either left or right-side post-surgical sessions independently. The parameter ∆VAS (change in VAS) could not be assessed due to a floor effect (i.e. scores of zero) in the pre-surgery VAS condition.

### 2.10 Inter-subject reliability of the CBF measurements

Inter-subject consistency of the ASL data was compared using an ICC approach previously described in the literature (Caceres et al., 2009). This was performed as a voxel-wise calculation of ICC, based upon the medians of ICC distributions (med ICC). We demonstrate the reliability of the pain network, whole GM volume, and targeted ROIs.

### 2.11 Inter- and intra-session reliability of the CBF measurements

Inter- and intra-session reliability of the ASL data was compared using an intra-voxel ICC measurement (ICC_v_) (Caceres et al., 2009; Raemaekers et al., 2007; Specht et al., 2003). This was calculated by extracting the CBF amplitudes of each voxel, and assessing the distribution of ICC values across voxels of each ROI (Caceres et al., 2009). Comparisons between the session pairs were used to assess inter-session reliability. For intra-session reliability, the CBF values of the first and third, and first and sixth pCASL scans were examined independently. These scans were chosen as they represent the start, mid-point, and end of the dynamic time-series, hence should reflect any temporal variations in CBF between the repeated measurements.

## 3 Results

### 3.1 Behavioural results

**Fig. 2:**
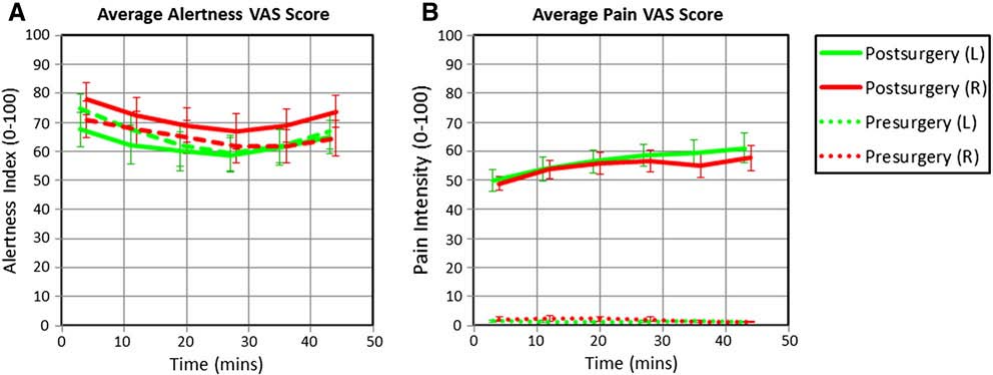
Concurrent VAS ratings of perceived alertness (A) and pain (B). Participants subjectively rated their experience following each of the six pCASL scans. Data represents the mean (± S.E.M.) of all subjects' ratings.

The VAS self-reported measures of alertness and pain are shown in Fig. 2. There were no significant differences in alertness between the pre- and post-surgical sessions (*p* = 0.35), indicating that voluntary attention was consistent across the group. Participants' subjective ratings of pain were significantly higher in the post-surgical sessions as compared to the pre-surgical sessions (*p* < 0.001). There were no significant differences in the VAS scores relating to the left or right third molar extraction (*p* = 0.97).

The ICC performance measures of alertness and pain VAS ratings demonstrated the highest reliability within-subjects. Both inter- and intra-session ICCs were consistently above 0.6 and 0.8 with a low coefficient of variation (CV), indicating that the test–retest reliability of the pain and alertness ratings was good-to-excellent. At the group level, inter-subject VAS ratings of alertness indicated a good level of reliability (ICC = 0.664). However, the pain ratings demonstrated only fair reliability between-subjects (ICC = 0.456), which indicates a significant contribution of pain state variability. The ICC results are summarised in Table 1.

### 3.2 Group-level inter-subject consistency of the CBF measurements

**Fig. 3:**
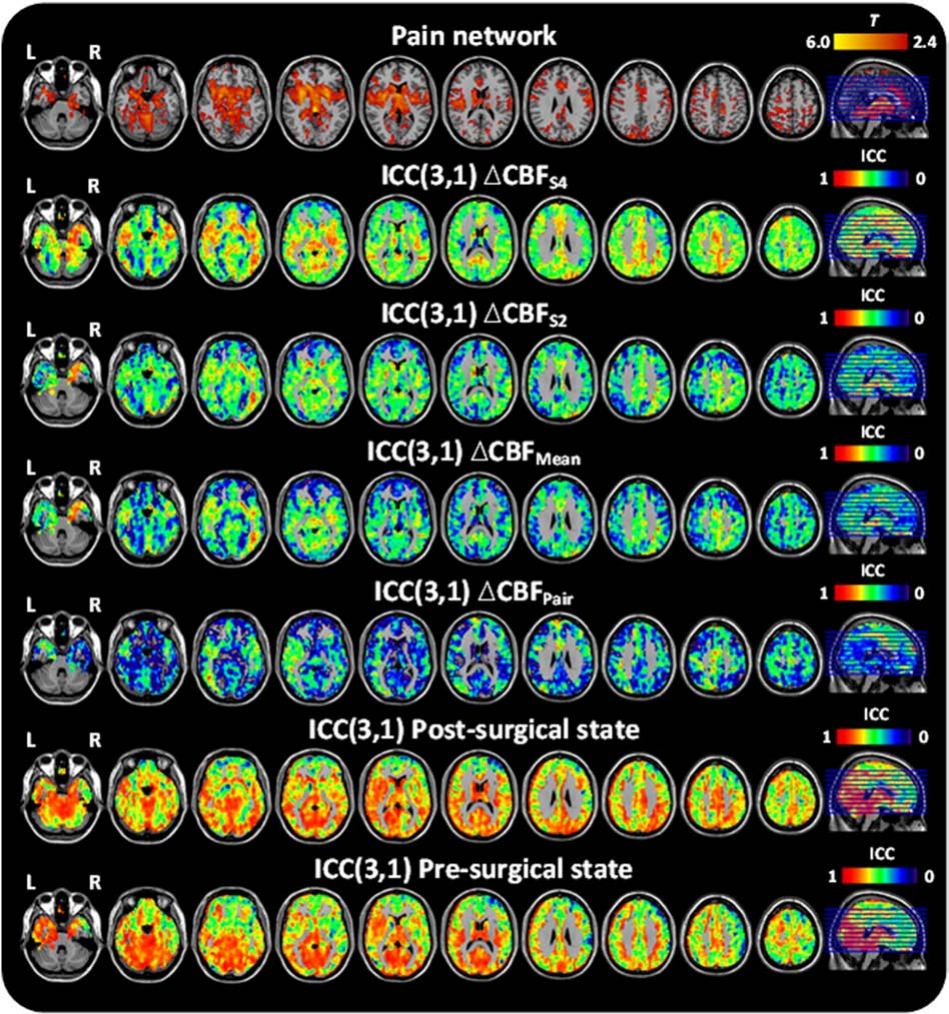
Group-level univariate and ICC analysis of pre- and post-surgical sessions, and ∆CBF.

**Fig. 4:**
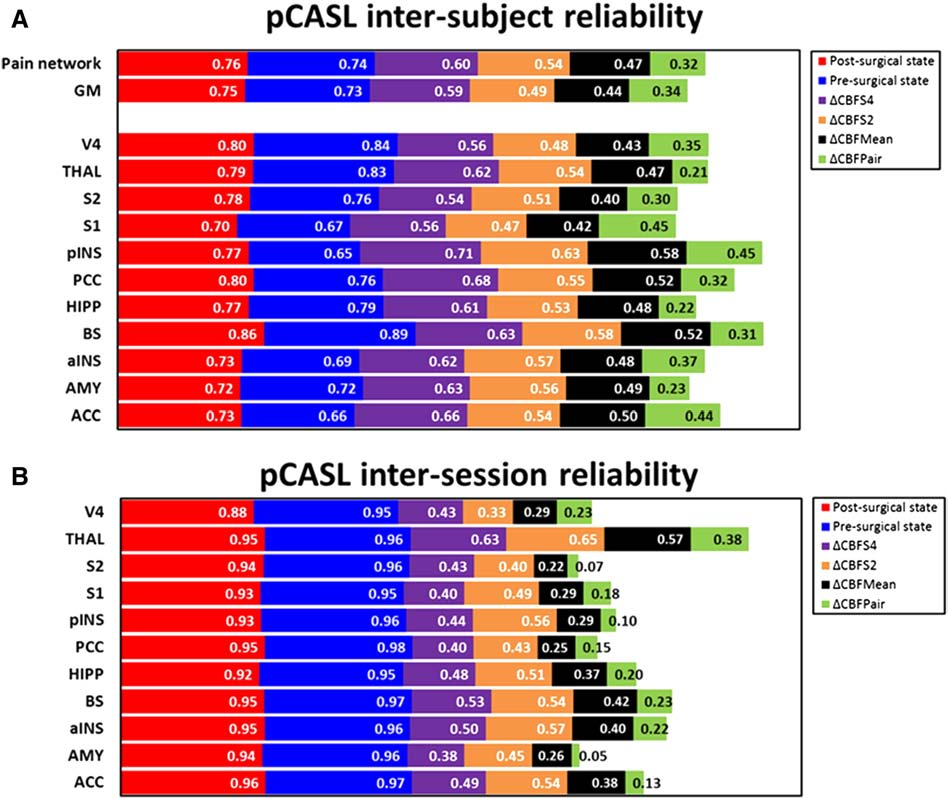
Inter-subject (A) and inter-session (B) reliability for the cortical grey-matter (GM), pain network, and targeted ROIs. Stacked columns represent the reliability magnitude including labels inside end. ICC values were calculated at a voxel-wise level. Abbreviations: amygdala (AMY), hippocampus (HIPP), brainstem (BS), thalamus (THAL), anterior insula (aINS), posterior insula (pINS), somatosensory cortex (primary, S1 and secondary, S2), posterior cingulate cortex (PCC), anterior cingulate cortex (ACC).

**Fig. 5:**
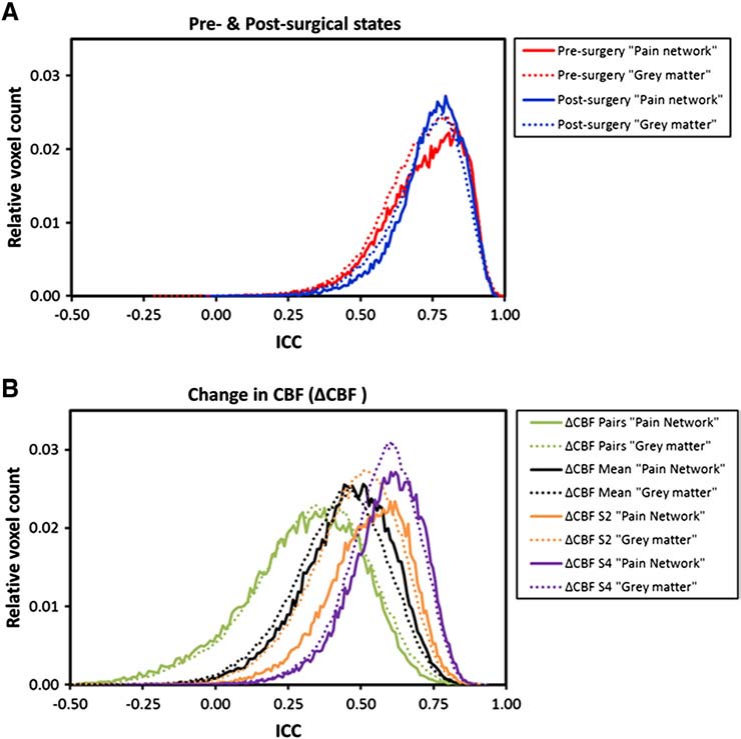
ICC distributions of the pre- and post-surgical states (A) together with the ∆CBF (change in CBF) (B). Plots show the relative number of activated voxels against ICC score for the grey-matter (dotted lines) and activated pain network (solid lines).

Univariate GLM analysis of the pre- and post-surgical sessions showed significant CBF increases in the respective anatomical target regions (Fig. 3) (see Supplementary information Table S1 for ROI values). Having confirmed that a network of rCBF increases is present during pain processing in the TME model, these data were used to assess the reliability of the pre- and post-surgical states together with the stability of the observed pain response (ΔCBF). The resulting ICC (3,1) maps for these conditions are depicted in Fig. 3. ICC values across the pre- and post-surgical states were high (0.763/0.746 and 0.744/0.731; [pain network/total GM]), which confirms high reliability across the individuals. Estimates of the reliability associated with the different ∆CBF calculations were less consistent: the between-subjects ICC was smallest in the ∆CBF_Pair_ (0.325/0.343), slightly higher using the mean of the two pre-surgical sessions (∆CBF_Mean_ 0.469/0.440), and greatest with the ∆CBF_S2_ (0.542/0.494) or ∆CBF_S4_ (0.604/0.589). The voxel-wise ICC values for individual ROIs can be found in Fig. 4A. Examining the ICC distributions, plots of the relative number of voxels against ICC score are shown in Fig. 5. The profiles of the pre- and post-surgical states (Fig. 5A) both demonstrate a pronounced negative skew in the ICC distribution, with the mass of the distribution concentrated on the right of the figure. There were relatively few low ICC values. For the parameter ΔCBF (Fig. 5B), the profiles of the four baseline calculation methods were considerably different. The negative skew was largest with ∆CBF_S2_ or ∆CBF_S4_, slightly smaller with the ∆CBF_Mean_, and smallest with the ∆CBF_Pair_ baseline. Importantly, in the ∆CBF_S2_ or ∆CBF_S4_ comparisons, voxels of the pain network were visibly more detached from the ICC values of the total GM volume.

Univariate GLM analysis of the pre- and post-surgical sessions showed significant CBF increases in the respective anatomical target regions (Fig. 3) (see Supplementary information Table S1 for ROI values). Having confirmed that a network of rCBF increases is present during pain processing in the TME model, these data were used to assess the reliability of the pre- and post-surgical states together with the stability of the observed pain response (ΔCBF). The resulting ICC (3,1) maps for these conditions are depicted in Fig. 3. ICC values across the pre- and post-surgical states were high (0.763/0.746 and 0.744/0.731; [pain network/total GM]), which confirms high reliability across the individuals. Estimates of the reliability associated with the different ∆CBF calculations were less consistent: the between-subjects ICC was smallest in the ∆CBF_Pair_ (0.325/0.343), slightly higher using the mean of the two pre-surgical sessions (∆CBF_Mean_ 0.469/0.440), and greatest with the ∆CBF_S2_ (0.542/0.494) or ∆CBF_S4_ (0.604/0.589). The voxel-wise ICC values for individual ROIs can be found in Fig. 4A. Examining the ICC distributions, plots of the relative number of voxels against ICC score are shown in Fig. 5. The profiles of the pre- and post-surgical states (Fig. 5A) both demonstrate a pronounced negative skew in the ICC distribution, with the mass of the distribution concentrated on the right of the figure. There were relatively few low ICC values. For the parameter ΔCBF (Fig. 5B), the profiles of the four baseline calculation methods were considerably different. The negative skew was largest with ∆CBF_S2_ or ∆CBF_S4_, slightly smaller with the ∆CBF_Mean_, and smallest with the ∆CBF_Pair_ baseline. Importantly, in the ∆CBF_S2_ or ∆CBF_S4_ comparisons, voxels of the pain network were visibly more detached from the ICC values of the total GM volume.

### 3.3 Within-subject inter-session reliability of the CBF measurements

Fig. 4B shows the regional inter-session ICC values for the pre- and post-surgical states together with the change in CBF (∆CBF). For the pre- and post-surgical states, a high level of agreement was found in all ROIs of the pain network. These voxel-based ICCs (ICC_v_) were consistently above 0.90 for each subject, demonstrating that the rCBF measurements have excellent inter-session reproducibility. By contrast, the ICC values for the ΔCBF images were much more varied with the ∆CBF_Pair_ and ∆CBF_Mean_ ranking poor-to-fair reliability, and ∆CBF_S2_ or ∆CBF_S4_ classified as fair to good.

### 3.4 Within-subject intra-session reliability of the CBF measurements

Intra-session reliability was reported for the post-surgical states. Sequential comparisons of the pCASL scans revealed that the voxel-based ICCs in all ROIs were consistently above 0.90 for every subject (irrespective of surgery-side) (Table 2). This suggests that the CBF measurements have excellent time-course reproducibility, and are stable from scan-to-scan.

## 4 Discussion

### 4.1 Summary

In the current literature there is very limited information available on the reliability of quantitative cerebral perfusion measures for the study of ongoing pain in experimental volunteers and patients. Here we present the test–retest analysis of concurrent pCASL and VAS measurements in a clinical model of on-going pain after third molar extraction (TME).

The key findings of this study are:

1) Within-subject, the inter- and intra-session reliability of the post-surgical pain state was ranked good-to-excellent across both pCASL and VAS modalities. The parameter ΔCBF (change in CBF between pre- and post-surgical states) performed reliably, provided that a single baseline condition (or the mean of more than one baseline) was used for subtraction.
2) Between-subjects, the pCASL measurements in the post-surgical pain state and ΔCBF were both characterised as reliable. However, the subjective VAS pain ratings demonstrated a significant contribution of pain state variability, which suggests diminished utility for interindividual comparisons.

### 4.2 Reliability at the behavioural level

Of the various methods for measuring pain, the visual analogue scale (VAS) is regarded the most sensitive. In the present study, inter- and intra-session reliability of VAS was consistently above 0.60, which indicates good-to-excellent levels of sensitivity to the changes in pain intensity within-subjects. As anticipated, the group-level pain scores demonstrated only fair reliability, reflecting a significant contribution of pain state variability. A likely reason for this numerical discrepancy is that the ICC measures are particularly sensitive to the small number of observations. One could argue that higher numbers of subjects may be required to detect a more robust behavioural response to pain. However, the VAS measures of alertness appeared not to suffer from this affect, suggesting that the variation in reliability could be explained by the influence of other contextual aspects of the patients' environment, which are known to separately influence pain perception (Tracey, 2010). A potential weakness of pain VAS is that each scale is one-dimensional and does not capture the full complexities of an individual's pain experience (Schiavenato and Craig, 2010). This remains a contentious issue in pain research (Davis et al., 2012; Robinson et al., 2013); however our paper focuses on the opportunities afforded through combining novel neuroimaging endpoints of pain with subjective self-report.

### 4.3 Group-level inter-subject consistency of the CBF measurements

Reliability and agreement are important issues in the conduct of clinical studies as they provide information about the amount of error inherent in any diagnosis, score, or measurement. In the present study, ICC values for the pre- and post-surgical states were characterised as good-to-excellent, while the reliability of ΔCBF ranged from poor-to-good depending on the method of ΔCBF calculation. These findings support the use of perfusion MRI measures for the study of on-going pain states and induced CBF responses. However, we demonstrate that measurement of more than one pre- and post-surgical CBF map has a profound effect on the reliability of the ΔCBF parameter.

ICC reliability indexes are not fixed characteristics of a measurement instrument. Factors associated with the study design (e.g. time-intervals between sessions and session order), the study cohort (e.g. age, gender, emotional status, and cognitive level), surgical interventions, etc., might all influence the magnitude of the variance between subjects as well as the error variance. To minimise the impact of these effects, we employed a counterbalanced within-subject study design, including strict inclusion and exclusion criteria as a means of establishing precision in the cohort. However, our reliability tests suggest that the cognitive or physiological contexts of the pre- and post-surgical states are not entirely independent or free of both functional and psychological interactions. Issues with *pure insertion* are common in studies that employ cognitive subtraction, and it is has been shown that factorial designs are generally more powerful in the analysis of cognitive processes (Friston et al., 1996). These effects were recently demonstrated by Klomp et al. (2012), who reported issues in detecting reliable drug-induced CBF changes with ASL using the test–retest method. With this in mind, we demonstrate that using a single baseline condition (or the mean of more than one baseline) may give more precise estimations of ICCs, and we suggest taking this innovation into account when designing future test–retest studies involving repeated measures, particularly in the context of a drug study.

We also observed that the high ICC values do not necessarily follow the high values of *t* (see Fig. 3). This discrepancy may originate from differences in the spatial distribution of the CBF response to pain, or from differences in intrinsic physiological factors between the individuals. Under normal resting conditions, perfusion has the potential to fluctuate considerably (Petersen et al., 2010) depending on the level of brain activity (Wenzel et al., 1996). Also, variations in blood T1, neuronal density or number, and arousal (Parkes et al., 2004) may cause individual differences in the perfusion estimate. Given that we carried out pCASL measurements at 3 T rather than 1.5 T, we had the advantage of longer T1, higher SNR, and improved spatial and temporal resolution. Uncertainties regarding the cerebrovascular kinetics or blood equilibrium magnetization might potentially bias the calculation of absolute CBF values; however, this would not affect the conclusions of the current paper regarding reliability of the on-going pain state. The ICC is clearly dependent on the heterogeneity of the sample and fluctuations in physiology induced by the pain state. We therefore conclude that any spatial non-uniformity of reliability in the CBF measurements may be driven by physiological variability rather than potential limitations of the pCASL technique. Further reliability studies in patient populations relevant for pain clinical trials will be important for the future use of ASL methodologies for assessing the cerebrovascular response to pain. Our results provide a framework for such assessments.

### 4.4 Within-subject inter-session reliability of the CBF measurements

**Fig. S2:**
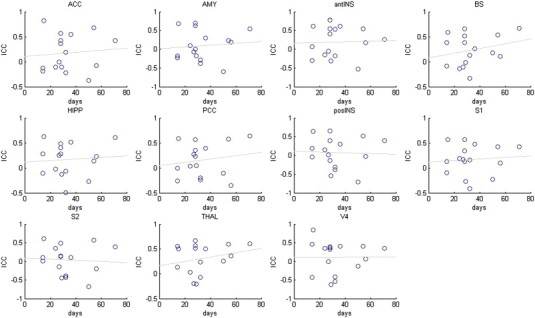
No significant correlations were observed between the repeated measures interval and within-subject ICC. Abbreviations: amygdala (AMY), hippocampus (HIPP), brainstem (BS), thalamus (THAL), anterior insula (antINS), posterior insula (posINS), somatosensory cortex (primary, S1 and secondary, S2), posterior cingulate cortex (PCC), anterior cingulate cortex (ACC).

Within-subject reliability is principally a longitudinal phenomenon. In the current study, the pre- and post-surgical states demonstrated excellent levels of reliability following a minimum two week interval in the TME model (see Fig. 4), which is comparable with previous studies into the longitudinal reliability of ASL in healthy volunteers (Gevers et al., 2009, 2011; Jain et al., 2012; Parkes et al., 2004; Wang et al., 2011) and neurological patients (Xu et al., 2010). The reliability of ΔCBF was acceptable depending on the method of the ΔCBF calculation. More specifically, the ICC values were smaller with ∆CBF_Pair_ and ∆CBF_Mean_ than with ∆CBF_S2_ or ∆CBF_S4_. We suggest that this highlights once again the inadequacy of the simple insertion model, which may be an intrinsic problem with testing reliability by the test–retest method at the individual subject level. It must be stressed that our study design did not allow us to perform the pre-surgical scans immediately before surgery, but were instead performed on different days. This limitation was considered when interpreting the results of this reliability assessment; however we found no relationship between interval length and ICC values (see Supplementary information — Fig. S2).

Within-subject reliability is principally a longitudinal phenomenon. In the current study, the pre- and post-surgical states demonstrated excellent levels of reliability following a minimum two week interval in the TME model (see Fig. 4), which is comparable with previous studies into the longitudinal reliability of ASL in healthy volunteers (Gevers et al., 2009, 2011; Jain et al., 2012; Parkes et al., 2004; Wang et al., 2011) and neurological patients (Xu et al., 2010). The reliability of ΔCBF was acceptable depending on the method of the ΔCBF calculation. More specifically, the ICC values were smaller with ∆CBF_Pair_ and ∆CBF_Mean_ than with ∆CBF_S2_ or ∆CBF_S4_. We suggest that this highlights once again the inadequacy of the simple insertion model, which may be an intrinsic problem with testing reliability by the test–retest method at the individual subject level. It must be stressed that our study design did not allow us to perform the pre-surgical scans immediately before surgery, but were instead performed on different days. This limitation was considered when interpreting the results of this reliability assessment; however we found no relationship between interval length and ICC values (see Supplementary information — Fig. S2).

There may also be intrinsic physiological differences in lateralisation of anatomy and/or function within-subjects. Initial assessments of lateralisation (Howard et al., 2011) revealed that the surgical pain appeared to have the same impact on each hemisphere, independent of whether the left or right third molar was removed. Bilateral activations in S1, S2, and the insular cortex have also been reported in two previous studies employing painful (Jantsch et al., 2005) and non-painful (Ettlin et al., 2004) dental stimulations. This has important implications for follow-up studies and crossover trials, as the ability to demonstrate low variation across repeated measures enables the detection of small alterations in CBF indices to monitor disease progression or the effect of therapeutic interventions. Other advantages of the ASL technique are that it is less invasive and less expensive than existing perfusion imaging approaches using radioactive tracers or paramagnetic contrast agents (Petersen et al., 2006). As ASL sequences become more widely used, evaluations of their reliability across the course of longitudinal studies will be important for understanding the advantages they offer in clinical pain research.

### 4.5 Within-subject intra-session reliability of the CBF measurements

Potential variability in the CBF measurements could be attributed to temporal variation. The temporal stability of the ASL signal was investigated with respect to the duration of scanning for each subject. Since the pCASL scans were repeated without repositioning, the potential error from aligning the acquisition and labelling plane was averted. Theoretically, this should minimise the operator-related variability, and begin to approach reproducibility values that are completely physiology dependent. As anticipated, the ICC values between pCASL scans were higher than those between sessions (Fig. 4 & Table 2), confirming that the CBF measurements within the on-going pain state have excellent time-course stability. The relative stability of these perfusion measurements to sustained temporal effects makes pCASL an attractive method to study naturalistic responses to pain. Furthermore, it allows within-subject investigations of spontaneous fluctuations in pain state, over relatively long-time intervals.

## 5 Conclusion

Here we present the test–retest analysis of concurrent pCASL and VAS measurements in a clinical model of on-going pain after third molar extraction (TME). Using ICC performance measures, we were able to quantify the reliability of the pain response and the on-going pain state, both at the group and individual case level. Within-subject, the inter- and intra-session reliability of the post-surgical pain state was characterised as good-to-excellent across both pCASL and VAS modalities. The parameter ΔCBF (change in CBF between pre- and post-surgical states) performed reliably, provided that a single baseline condition (or the mean of more than one baseline) was used for subtraction. Between-subjects, the pCASL measurements in the post-surgical pain state and ΔCBF were both characterised as reliable. However, the subjective VAS pain ratings demonstrated a significant contribution of pain state variability, which suggests diminished utility for interindividual comparisons. These analyses indicate that the pCASL imaging technique has considerable potential for the comparison of within- and between-subjects differences associated with pain-induced state changes and baseline differences in regional CBF. They also suggest that differences in baseline perfusion and functional lateralisation characteristics may play an important role in the overall reliability of the estimated changes in CBF. Repeated measures designs have the important advantage that they provide good reliability for comparing condition effects because all sources of variability between subjects are excluded from the experimental error. The ability to elicit reliable neural correlates of on-going pain using quantitative perfusion imaging might help support the conclusions derived from subjective self-report.

The following are the supplementary data related to this article.

Supplementary material.

**Table S1:**
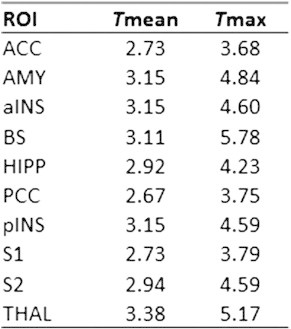
CBF main effects in the regions-of-interest across pain vs no pain sessions. All reported effects correspond to a threshold of *p* < 0.01 (*t* = 2.41) cluster-corrected. Abbreviations: *T*_mean_, effect size for mean of the total ROI data; *T*_max_, effect size for the peak voxel in the ROI.

Supplementary data to this article can be found online at http://dx.doi.org/10.1016/j.nicl.2013.09.004.

## Conflict of interest

The collection of the data was funded by Pfizer Global Research and Development UK. MAH and KK were paid on grant income from this source. JPH and WV were employees of Pfizer. DJH was paid with grant income from the MRC.

## Figures and Tables

**Table 1:** Reliability measures for the subjective behavioural ratings of pain and alertness. ICC, intraclass correlation coefficients; CV, coefficient of variation.

VAS reliability								
Visual analogue scales	Inter-subject		Inter-session		Intra-session			
			Left vs right		Left		Right	
	ICC	CV	ICC	CV	ICC	CV	ICC	CV
Pain intensity	0.456	0.285	0.602	0.200	0.830	0.300	0.861	0.267
Alertness	0.664	0.359	0.640	0.203	0.800	0.390	0.940	0.320

**Table 2:** Intra-session reliability of the representative pain ROIs. ICC values are compared between first and third, and first and sixth pCASL scans in the post-surgical pain states (ICC_v_; the intra-voxel reliability; SEM, standard error from measurement).

pCASL intra-session reliability								
ROI	Left-side post-surgical state				Right-side post-surgical state			
	pCASL 1 vs 3		pCASL 1 vs 6		pCASL 1 vs 3		pCASL 1 vs 6	
	ICC_v_	SEM	ICC_v_	SEM	ICC_v_	SEM	ICC_v_	SEM
ACC	0.965	0.006	0.962	0.006	0.968	0.003	0.966	0.006
AMY	0.937	0.009	0.921	0.017	0.944	0.004	0.938	0.007
alNS	0.967	0.005	0.959	0.004	0.970	0.005	0.964	0.004
BS	0.974	0.003	0.970	0.004	0.974	0.003	0.970	0.002
HIPP	0.931	0.008	0.923	0.010	0.938	0.004	0.938	0.004
PCC	0.974	0.007	0.973	0.007	0.977	0.005	0.972	0.006
pINS	0.958	0.005	0.951	0.007	0.963	0.003	0.961	0.005
S1	0.957	0.004	0.952	0.007	0.953	0.008	0.947	0.007
S2	0.974	0.004	0.968	0.003	0.976	0.003	0.971	0.004
THAL	0.955	0.006	0.945	0.012	0.957	0.005	0.955	0.012
