# Supplementary material for: Quantifying the test–retest reliability of cerebral blood flow measurements in a clinical model of on-going post-surgical pain: A study using pseudo-continuous arterial spin labelling
Source: Neuroimage Clin. 2013 Sep 16;3:301–10. doi: 10.1016/j.nicl.2013.09.004 (PMC3797555; doi:10.1016/j.nicl.2013.09.004)
Supplement: Supplementary file 1 — Supplementary material. [file mmc1.docx]

**Pseudo-continuous arterial spin labeling & absolute quantification of CBF**

Participants were scanned in a General Electric Signa HDX 3 Tesla scanner at the Centre for Neuroimaging Sciences, Institute of Psychiatry, London. rCBF measurements were made using a pseudo-continuous arterial spin labeling technique (pCASL) [[1](#_ENREF_1)]. Arterial blood was labelled using a 1.5s train of Hanning radio frequency (RF) pulses of 500μs duration, with an inter-pulse period (*τ*) of 1500μs. The amplitude of each Hanning RF pulse was 8.4μT, (effective flip angle of 32.3 degrees). Gradient pulses of the same duration and repetition rate (each followed by a refocusing lobe) accompanied the RF train to achieve flow-driven adiabatic inversion. In the control phase, the gradient lobes were of equal area but in the labeling phase, the gradient lobes were unbalanced, in order to achieve a net gradient amplitude (*G_ave_* ) of 0.7mT/m. The maximum gradient amplitude under the Hanning pulses was 7mT/m. These values satisfy the adiabatic condition for inversion and place the first aliased labeling plane away from the excitation bandwidth of the Hanning pulse [[1](#_ENREF_1)]. In the control phase, the sign of alternate Hanning pulses was reversed and since the gradient lobes are of equal area, the control pulse compensates for magnetization transfer effects of the labeling pulse while achieving no net inversion of arterial spins. In the labeling phase however, the phase of each Hanning pulse was incremented by a small fraction given by the product:

$$\gamma G_{ave} \tau d$$

where *γ* is the gyro-magnetic ratio of ^1^H spins, *G_ave_* is the average net gradient over the labeling pulse, *τ* is the pulse-to-pulse period and *d* is the distance from the labeling plane to the iso-centre. This distance was selected from the localizer scan and standardized on all subjects to be exactly 1cm above the most inferior point of the cerebellum. The distance from the labeling plane to the bottom of the imaging slab was approximately 1cm. By default, the mid-point of the imaging slab always coincided with the magnet iso-centre.

We employed a post-labeling delay of 1.5s, this delay has been found to be appropriate to minimize vascular artefacts [[1](#_ENREF_1), [2](#_ENREF_2)] and has been employed successfully in several studies that utilized a single TI ASL method in healthy volunteers [[3-8](#_ENREF_3)]. The image was acquired with a 3D Fast Spin Echo (FSE) spiral multi-slice readout. To minimize blurring, the spiral acquisition was very short (4ms), and the required resolution was achieved with 8 spiral interleaves (effective TE 32ms/TR = 5500ms; Echo Train Length (ETL) = 64). Images were acquired at a 48x64x60 matrix on a 18x24x18cm field of view and reconstructed to a 256^2^ in plane matrix, resulting in a nominal spatial resolution of 1x1 x 3mm. For signal averaging purposes, three pairs of tagged-untagged images were collected in succession. Selective saturation of the image slab was applied at 4.3s before acquisition, selective inversion was applied 3s before acquisition with further non-selective inversions at 1.5s, 764ms, 334ms and 84ms before imaging. This repeated inversion achieved successful suppression of the background static tissue signal, thus maximizing the sensitivity to blood perfusion [[9](#_ENREF_9)].

Following the three arterial spin labeling (ASL) control-label pair averages, reference images were acquired with the same imaging sequence but with inversion recovery preparation instead of ASL. One sequence with saturation of 4.3s and then an inversion at 1650ms before imaging was used to create a fluid suppressed image. A second sequence with saturation at 4.3s and then inversion at both 2408ms and 511ms was also acquired to create a fluid and white matter suppressed image. For both these sequences, the receiver gain was automatically lowered by 21 dB relative to the ASL sequence to avoid receiver saturation. These images were used to quantify blood flow from the mean perfusion weighted difference image computed from the three tagged-untagged pairs, as described below. The sensitivity of the image was also calibrated to water at each voxel in order to account for the relative sensitivity of the coil elements [[2](#_ENREF_2), [10](#_ENREF_10), [11](#_ENREF_11)]. By means of a neighborhood maximum algorithm to avoid regions with partial volume of suppressed fluid, a low-resolution sensitivity map was created. This map was calibrated for water sensitivity by assuming the tissue was predominantly white matter with a water concentration of 0.735 gm/ml [[12](#_ENREF_12)] and a T1 of 900ms, and using the equations for inversion recovery signal attenuation. This calibration produced a sensitivity map, C, equal to the fully relaxed MRI signal intensity produced by one gm of water per ml of brain. It is worth noting that assuming pure grey matter produces only a 5% calibration difference. With this co-registered sensitivity map C, CBF was calculated at each voxel using the equation:

$$CBF=\frac{\rho_{b}\left(S_{c}-S_{l}\right)}{2\alpha C \omega_{a}T1_{a}\exp\left(-\frac{w}{T1_{a}}\right)\left(1-\exp\left(-\frac{tl}{T1_{a}}\right)\right)}$$

where *ρ_b_* is 1.05g/ml (the density of brain tissue; [[12](#_ENREF_12)]), α is the labeling efficiency (assumed to be 95% for labeling times 75% for background suppression; [[13](#_ENREF_13)], *w* is 1.5s (the post-labeling delay; [[2](#_ENREF_2)]), *tl* is 500ms (the labeling duration), T1_a_ is 1.4 ms (the T1 of arterial blood which was slightly lower than the value of [[14](#_ENREF_14)]), *ω_a_* 0.85 g/ml (the density of water in blood;[[12](#_ENREF_12)]), *S_l_* and *S_c_* are the signal intensities in the labeled and control images, respectively.

The whole ASL pulse sequence, including the acquisition of calibration images, was performed in 6:08min and repeated 6 times in succession during the scan. A conventional T2 weighted FSE image was acquired for co-registration and normalization and a fast spin echo fluid-attenuated inversion-recovery (FLAIR FSE) scan was made in order to exclude the presence of pathology.

**References**

1. Dai, W., et al., *Continuous flow-driven inversion for arterial spin labeling using pulsed radio frequency and gradient fields.* Magn Reson Med, 2008. **60**(6): p. 1488-97.

2. Alsop, D.C. and J.A. Detre, *Reduced transit-time sensitivity in noninvasive magnetic resonance imaging of human cerebral blood flow.* J Cereb Blood Flow Metab, 1996. **16**(6): p. 1236-49.

3. El-Hage, W., et al., *Resting-state cerebral blood flow in amygdala is modulated by sex and serotonin transporter genotype.* Neuroimage, 2013. **76**: p. 90-7.

4. Howard, M.A., et al., *Alterations in resting-state regional cerebral blood flow demonstrate ongoing pain in osteoarthritis: An arterial spin-labeled magnetic resonance imaging study.* Arthritis Rheum, 2012. **64**(12): p. 3936-46.

5. Howard, M.A., et al., *Beyond patient reported pain: perfusion magnetic resonance imaging demonstrates reproducible cerebral representation of ongoing post-surgical pain.* PLoS One, 2011. **6**(2): p. e17096.

6. Towgood, K.J., et al., *Regional cerebral blood flow and FDG uptake in asymptomatic HIV-1 men.* Hum Brain Mapp, 2012.

7. Marquand, A.F., et al., *Dissociable effects of methylphenidate, atomoxetine and placebo on regional cerebral blood flow in healthy volunteers at rest: a multi-class pattern recognition approach.* Neuroimage, 2012. **60**(2): p. 1015-24.

8. Handley, R., et al., *Acute effects of single-dose aripiprazole and haloperidol on resting cerebral blood flow (rCBF) in the human brain.* Hum Brain Mapp, 2013. **34**(2): p. 272-82.

9. Maleki, N., W. Dai, and D.C. Alsop, *Optimization of background suppression for arterial spin labeling perfusion imaging.* MAGMA, 2012. **25**(2): p. 127-33.

10. Buxton, R.B., et al., *A general kinetic model for quantitative perfusion imaging with arterial spin labeling.* Magn Reson Med, 1998. **40**(3): p. 383-96.

11. Williams, D.S., et al., *Magnetic resonance imaging of perfusion using spin inversion of arterial water.* Proc Natl Acad Sci U S A, 1992. **89**(1): p. 212-6.

12. Herscovitch, P. and M.E. Raichle, *What is the correct value for the brain--blood partition coefficient for water?* J Cereb Blood Flow Metab, 1985. **5**(1): p. 65-9.

13. Garcia, D.M., G. Duhamel, and D.C. Alsop, *Efficiency of inversion pulses for background suppressed arterial spin labeling.* Magn Reson Med, 2005. **54**(2): p. 366-72.

14. Lu, H., et al., *Determining the longitudinal relaxation time (T1) of blood at 3.0 Tesla.* Magn Reson Med, 2004. **52**(3): p. 679-82.
